# Effective Recellularization Using Mesenchymal Stem Cell Monoculture for Next-Generation Heart Valves

**DOI:** 10.3390/bioengineering13050546

**Published:** 2026-05-11

**Authors:** So Young Kim, Ja-Kyoung Yoon, Serin Kim, Sunhi Ko, Yerin Shin, Gi Beom Kim, Hong-Gook Lim, Yong Jin Kim

**Affiliations:** 1 Department of Thoracic and Cardiovascular Surgery, Seoul National University Hospital, Seoul National University College of Medicine, 101 Daehak-ro, Jongno-gu, Seoul 03080, Republic of Korea; 2 Department of Pediatrics, Samsung Medical Center, 81 Irwon-ro, Gangnam-gu, Seoul 06351, Republic of Korea; 3 Department of Pediatrics, Seoul National University Hospital, Seoul National University College of Medicine, 101 Daehak-ro, Jongno-gu, Seoul 03080, Republic of Korea; 4 Department of Thoracic and Cardiovascular Surgery, Sejong General Hospital, 28 Hohyeon-ro 489beon-gil, Bucheon-si 14754, Gyeonggi-do, Republic of Korea

**Keywords:** tissue engineering, biomaterial, xenograft, decellularization, recellularization

## Abstract

Objective: Effectively eliminating xenoimmunogenicity and achieving recellularization in cardiac xenografts remains a critical challenge in developing an ideal implantable xenograft. We have previously demonstrated that the removal of major antigens, including Galα1-3Gal (α-Gal) epitope and non-human sialic acid N-glycolylneuraminic acid (Neu5Gc), using α-galactosidase and peptide N-glycosidase F (PNGase-F), enables a synergistic effect with decellularization, significantly reducing the expression of carbohydrate-binding lectins without altering the biomechanical properties of the graft. The aim of this study was to establish an effective method for in vitro recellularization by seeding human mesenchymal stem cells (MSCs) on decellularized cardiac xenografts that had undergone optimal xenoantigen removal using α-galactosidase and PNGase-F. Additionally, this study aimed to evaluate the potential for in vivo recellularization. Methods: Decellularized porcine pericardium scaffolds treated with both enzymes were further modified by forming a fibrin mesh on their surface and within their structure, followed by the attachment of heparin and human vascular endothelial growth factor to the mesh. Subsequently, the scaffolds were seeded with human adipose tissue-derived stem cells for 8 weeks. In vitro recellularization, differentiation, and extracellular matrix remodeling of decellularized and enzyme-treated xenografts were assessed using vimentin, calponin, fibronectin, CD31, VWF, and phalloidin staining. To evaluate the potential for in vivo recellularization, decellularized glutaraldehyde-crosslinked xenografts with anticalcification treatments were seeded with rat bone marrow MSCs and implanted into rats subcutaneously to evaluate cell infiltration and calcification via histology, von Kossa staining, and micro-computed tomography. Results: In decellularized xenografts treated with both enzymes, stronger signals were detected and mesenchymal cell infiltration into the tissue was significantly faster, leading to accelerated recellularization. This recellularization process was more pronounced as time went on, with greater cell infiltration and evidence of cell differentiation. An in vivo study showed that decellularization and anticalcification treatments revealed stronger vimentin staining in histological analysis. The recellularization for our biocompatible scaffolds exhibited a lower degree of calcification compared to the non-recellularized tissue. Conclusions: We successfully developed major xenoantigen-free scaffolds by demonstrating the safety and synergistic effect of α-galactosidase and PNGase-F treatments and proved, for the first time, the effectiveness of recellularization using a human MSC monoculture on xenoantigen-free scaffolds. Furthermore, there was potential for in vivo recellularization of our biocompatible scaffolds seeded with MSCs.

## 1. Introduction

The development of biocompatible and durable heart valve substitutes remains a major challenge in cardiovascular tissue engineering, particularly for pediatric patients with congenital heart disease. Decellularized xenografts provide promising scaffolds, but their application is hindered by immunogenicity, limited regenerative capacity, and susceptibility to calcification. We believe that these limitations can be addressed through tissue recellularization, and have explored diverse tissue engineering approaches to design valves that exhibit minimal immunogenicity in humans, reduced propensity for thrombosis and calcification, robust durability, and the potential for growth [1]. To date, our research team has pursued xenograft-based studies, with a primary focus on refining and optimizing the decellularization process [2,3,4,5,6]. In this process, we have investigated and published various methods to reduce cellular remnants and immunogenicity without damaging the tissue structure. As a result, we developed and commercialized the PULSTA valve by processing porcine pericardium into a functional heart valve [7,8,9].

We have previously demonstrated that the removal of major antigens, the Galα1-3Gal (α-Gal) epitope and the non-human sialic acid N-glycolylneuraminic acid (Neu5Gc) using α-galactosidase and peptide N-glycosidase F (PNGase-F) enables a synergistic effect with decellularization, significantly reducing the expression of carbohydrate-binding lectins without altering the graft’s biomechanical properties [2]. Our team hypothesized that going beyond decellularization to achieve recellularization with autologous cells could produce tissues with greater durability and remodeling potential. Based on this hypothesis, we have previously reported the results of recellularizing xenoantigen-free decellularized cardiac xenografts. In our previous study, we successfully achieved in vitro tissue recellularization by performing decellularization that simultaneously removed both α-Gal and Neu5Gc xenoantigens, followed by co-culturing with human mesenchymal cells and human umbilical vein endothelial cells (HUVEC) [10].

However, considering the potential future application of the construct as a functional heart valve tissue, this study aimed to determine whether meaningful recellularization could be achieved using a mesenchymal cell monoculture for more practical and clinically relevant use, and establish a clinically translatable recellularization strategy for decellularized porcine pericardial scaffolds by combining dual enzymatic xenoantigen removal and human adipose-derived mesenchymal stem cell (hADSC) seeding, without the need for endothelial co-culture.

## 2. Materials and Methods

### 2.1. Tissue Preparation of Porcine Pericardia

Porcine pericardia were isolated from the heart and were obtained from Taewoong Medical Inc. (Gimpo-si, Republic of Korea). For tissue preparation, porcine pericardium was rinsed in ice-cold phosphate normal saline with 1% antibiotic/antimycotic. The fat and connective tissue were trimmed. The tissues were immersed in 0.1% PAA and 4% ethanol in distilled water for 4 h, and then washed with buffered saline solution (PBS) under agitation at room temperature (RT). Some pericardia were analyzed as untreated specimens for DNA content after sectioning for histology.

### 2.2. Decellularization and Glutaraldehyde Fixation

The porcine pericardium was directly decellularized using 0.25% sodium dodecyl sulfate (SDS) (Sigma-Aldrich, St. Louis, MO, USA, 436143) in diH_2_O containing 0.5% (*v*/*v*) Triton X-100. For decellularization, each process using various solutions was carried out under an agitator (180 rpm) in an orbital shaker (SHO-2D, DAIHAN).

The porcine pericardial tissues were initially decellularized with a hypotonic solution with 0.25% SDS for 24 h at 4 °C and washed with distilled water for 1 h. Then they were treated with a hypotonic solution containing 0.5% Triton X-100 for 24 h at 4 °C, and then washed with distilled water for 12 h at 4 °C. After washing, these tissues were treated with an isotonic solution for 48 h at 4 °C. The tissues were treated in PBS with 30% polyethylene glycol 1000 (PEG-1000) and 1% antibiotic/antimycotic for 48 h at 4 °C. The tissues were finally treated with a hypertonic solution for 3 h at 4 °C, and then these tissues were washed in PBS. Decellularized pericardial tissues were obtained using our established protocol [4].

For in vivo studies after recellularization, the porcine pericardial tissues were prepared into four groups: native, native + 0.5% glutaraldehyde (GA), decellularized, and decellularized + 0.5% GA. Tissues were fixed in PBS-buffered 0.5% GA solution (pH 7.4) at RT for 14 days (0.5% GA). Tissue was fixed in 0.5% GA for 72 h, and then fixed in 0.25% GA with 75% ethanol and 5% octanol for 48 h, and then the tissues were again treated in 0.25% GA for 7 days and then in 0.2 M glycine solution for 48 h (decellularized + 0.5% GA).

### 2.3. Enzyme Digestion

After decellularization, porcine pericardia were treated with PNGase-F (#P0704L, 1000U, NEB) in concentrations of 2000 unit/mL, whereas recombinant α-galactosidase (G7163, sigma) was treated with concentrations of 0.1 unit/mL. In addition, SDS + Tx-decellularized porcine pericardia were treated with α-galactosidase in combination with PNGase-F. Native pericardial tissues served as the untreated control or the negative control. Porcine pericardial tissues (1 × 1 cm size) with isotonic solution were applied for 24 h at 4 °C with agitation. Each enzymatic treatment with sliced tissues was performed at 37 °C for 24 h under agitation. After treatment, sliced tissues were washed in PBS under agitation (120 rpm; 10 min each; 3 times washes) at RT. Finally, the tissue samples were stored in sterile PBS supplemented with 1% (*v*/*v*) penicillin/streptomycin and 400 μL/L amphotericin B for 24 h at 4 °C.

### 2.4. Histology and Quantification of DNA Contents

Native and decellularized porcine pericardia were treated with different enzymatic treatments: SDS + Tx-decellularized tissues were treated with α-galactosidase, PNGase-F, and α-galactosidase in combination with PNGase-F or no enzyme. Dissected samples of native, decellularized, and both decellularized and enzymatically treated pericardia were fixed in 4% paraformaldehyde for 24 h and embedded in paraffin. Histological tissue sections (5 μm) of each sample were stained for hematoxylin and eosin (H&E) and immunofluorescence (IF) staining. Sliced samples of native and decellularized tissue for DNA quantification were homogenized, and then ground tissue samples (100 mg) were incubated in lysis buffer with proteinase K and RNase A solution for 24 h at 56 °C for 30 min. The QiAmp DNA Mini kit (Qiagen, Hilden, Germany) was used to measure DNA content according to the manufacturer’s instructions. Isolated DNA pellets were determined using a NanoDrop UV-VIS spectrophotometer (Thermo Fisher), and the absolute amount of DNA (ng) was quantified in relation to the total tissue mass (mg) used.

### 2.5. Cell Culture of Human Adipose Tissue-Derived Stem Cells (hADSCs)

Human adipose-derived stem cells (hADSCs) were purchased from Thermo Scientifics (R7788115). The hADSCs were cultured at 37 °C in a humidified atmosphere of 5% CO_2_ with Dulbecco’s Modified Eagle Medium (DMEM; LM001-05, Welgene, Gyeongsan, Republic of Korea) medium containing 10% fetal bovine serum (FBS), 1% penicillin/streptomycin (P/S). Rat bone marrow-derived mesenchymal stromal cells (BM-MSCs) were purchased from (Cell Biologics (RA-6043; Cell Biologics, Chicago, IL, USA). The BM-MSCs were cultured at 37 °C in a humidified atmosphere of 5% CO_2_ with Minimum Essential Medium Eagle, Alpha (α-MEM, Welgene, LM 008-01) medium containing 10% FBS, 1% P/S. The cells were grown in T-75 flasks for 5 to 7 days, and they were kept within 5 to 6 passages for the experiments. The cell culture medium of both cells was changed every 3 days.

After cell seeding on fabricated scaffolds, all pericardial scaffolds used in these experiments were cultured in DMEM or α-MEM, supplemented with 2% FBS, 1% P/S, transforming growth factor beta 1 (TGF-β1, 2.5 ng/mL, ab50036, Abcam), and bone morphogenetic protein 4 (BMP-4, 2.5 ng/mL, SRP6156, Sigma).

### 2.6. Fibrin Mesh Fabrication for Pericardial Scaffolds Modification

For recellularization of xenoantigen-free cardiac scaffolds, modification of porcine pericardia was performed in five experimental conditions: native, decellularized, decellularized + α-galactosidase (0.1 unit/mL), decellularized + PNGase-F (2000 unit/mL), and α-galactosidase in combination with PNGase-F. For in vivo studies after recellularization, the porcine pericardial tissues were modified into four groups: native, 0.5% GA, decellularized, and decellularized + 0.5% GA. The fibrin mesh was created by combining a solution containing fibrinogen, thrombin, and heparin, according to each process. The pericardial scaffolds were cut into approximately 1 × 1 cm (length × width, n = 3), and sliced samples were sterilized using 70% alcohol. Prior to cell seeding, the sterile scaffolds were washed using sterilized PBS, and the sliced pericardial scaffolds were fabricated with a fibrin mesh (P- or DP-Fb) in a 12-well plate. The scaffolds were washed using 0.05 M Tris-HCl buffer (TB) solution, which was modified by attachment of heparin (P- or DP-Fb + H) overnight at 4 °C. Upon overnight incubation, the fibrin mesh + heparin (P- or DP-Fb + H) scaffolds were washed with sterilized PBS, and were incubated with vascular endothelial growth factor (VEGF 165, 100 ng/mL in PBS, GeneScript USA Inc.) for 2 h at RT and washed with PBS, using an established protocol [11].

### 2.7. Human Adipose-Derived Stem Cells Seeded onto Modified Pericardial Scaffolds

For cellular infiltration of hADSCs into the fibrin mesh-coated scaffolds, all pericardial scaffolds were spread on both sides and were seeded onto the central region. However, hADSCs were not seeded on some modified pericardial scaffolds, which were used as a negative control (n = 3). The hADSCs (passage 5) were seeded on the upper side of all modified pericardial scaffolds (P- or DP-Fb + H + VEGF) at a density of 5 × 10^4^ cells (day 0) and per well in a 12-well plate. The seeded scaffolds were turned over, and hADSCs were seeded on lower side of scaffolds at a density of 5 × 10^4^ cells (day 0) in DMEM medium supplemented with 2% FBS, transforming growth factor beta 1 (TGF-β1, 2.5 ng/mL, ab50036, Abcam), and bone morphogenetic protein 4 (BMP-4, 2.5 ng/mL, SRP6156, Sigma), and these pericardial scaffolds were cultured until day 56. The rat BM-MSCs (passage 5) were seeded on top of each modified pericardial scaffold (P- or DP-Fb + H + VEGF) at a density of 1 × 10^5^ cells (day 0) in 50 μL culture medium with 10% FBS for 3 h at 37 °C and 5% CO_2_. The seeded scaffolds were turned over, and the cells were seeded on the bottom of the scaffolds at a density of 1 × 10^5^ cells (day 0) using 50 μL α-MEM medium in a 12-well plate. After cell attachment to the scaffolds, 2 mL culture media was added to each cell-seeded scaffold, and then the scaffolds were cultured for 3 days.

### 2.8. Immuno-Fluorescence Staining of Recellularized Pericardial Scaffolds

After 56 days of in vitro culturing, the seeded scaffolds were cut in half, fixed in 4% para-formaldehyde solution, and processed for H&E and IF staining. IF staining was performed using embedded sections and deparaffinized sections. For the IF staining, all scaffolds were incubated in antigen retrieval buffer (10 mM citrate buffer, pH 6.0) at 95 °C for 10 min, followed by 3 washes in PBS for 5 min each. The samples were then incubated in 5% blocking serum for 60 min at RT. The seeded and unseeded scaffold samples were then incubated with primary antibodies for vimentin (ab16700, Abcam, dilution 1:200), calponin (ab227661, Abcam, dilution 1:100), fibronectin (F0916, sigma, dilution 1:100), and CD31 (ab182981, Abcam, dilution 1:2000) at 4 °C overnight. Upon overnight incubation, all scaffolds were then washed 3 times for 10 min each in PBS-T, and incubated for 2 h at RT with anti-rabbit secondary antibody conjugated with Alexa Fluor 488 (A11008, Invitrogen, Carlsbad, CA, USA; 1:500) and anti-mouse secondary antibody conjugated with Alexa Fluor 488 (AB150113, Invitrogen, 1:500). The samples were then washed with PBS-T for 10 min. Nuclei were counterstained with 4ʹ,6-diamidino-2-phenylindole (DAPI) (Invitrogen, 1:1000) for 1 min. The sections were washed 3 times with PBS-T and mounted using Shandon^TM^ Immuno-Mount (Thermo Fischer Scientific). The stained sections were imaged using an inverted fluorescence microscope (DMI4000B, Leica Microsystems, Wetzlar, Germany). Merging of the stained images and DAPI was processed through Application Suite X Image Viewer software version 3.5.7 (Leica), and cell counting was performed using the ImageJ/FIJI software (NIH, Bethesda, MD, USA), version 1.54f (FIJI distribution).

### 2.9. Immuno-Fluorescence Using Confocal Microscopy

All scaffolds were fixed in 4% paraformaldehyde solution and processed for confocal microscopy. Paraffin-embedded sections were incubated in 0.2% Triton-X for 15 min, and then the sections were washed 3 times with PBS-T. After washing in PBS-T, the samples were then incubated in 5% blocking serum for 60 min at RT. The sections were incubated overnight at 4 °C with primary antibodies directed against von Willebrand factor (F8/86) (MA5-14029, Thermo Fischer Scientific, dilution 1:50). After incubation, the sections were further incubated with goat anti-rabbit secondary antibody conjugated with Alexa Fluor 488 (A11008, Invitrogen, 1:500) for 1 h at RT, and then phalloidin-647 (ab176759, Abcam, dilution 1:1000) for 30 min at RT. After the animal experiment, confocal analysis of harvested tissue sections was conducted. BM-MSC seeded sections were incubated with vimentin-647 (ab176759, Abcam, dilution 1:1000) for 30 min at RT. Nuclei were counterstained with DAPI (Invitrogen, 1:1000) for 1 min. The stained sections were analyzed by confocal microscopy (STELLARIS5, Leica).

### 2.10. Animal Experiments

Four-week-old male Sprague-Dawley rats (n = 20) were purchased from the KOATECH Co., Ltd. All procedures were carried out in accordance with the Guiding Principles on Care and Use of Animals of Seoul National University Hospital (IACUC No.24-00181-S1A0). For recellularized in vivo experiments, anesthesia was performed by intraperitoneal (i.p) injection with a mixture of Zoletil 50 25 mg/kg and xylazine 10 mg/kg. After the rats were anesthetized and shaved, six subcutaneous pouches were created in the dorsal area of each animal. Each group had its sliced scaffold samples (6 × 6 mm each) implanted into each pouch, and the wounds were closed with 6/0 nylon sutures. Native and decellularized porcine pericardial scaffolds with or without 0.5% GA, in the absence or in the presence of seeded BM-MSC, were implanted intradermally in a rat dorsal skin and explanted after 7 and 28 days. The animals were divided into 4 groups: native (n = 12), 0.5% glutaraldehyde (n = 12), decellularized (n = 12), and 0.5% GA-decellularized (n = 12). After each implantation, a papule was observed at the implantation site in the dorsal area of each animal. Cell-seeded scaffold-implanted rats were kept in a light/dark cycle until the next experiment. Four weeks after implantation, the scaffold samples were harvested, and finally, the implanted scaffolds were harvested on day 28. Upon harvest, examination of histological features and calcification using the harvested scaffolds was conducted.

### 2.11. Von Kossa Calcium Staining of Scaffolds After Implantation

Histological paraffin sections (5 μm) of each sample were stained for hematoxylin and eosin (H&E) and Von Kossa staining. Von Kossa staining was used to examine the calcium deposition in the implanted samples. The tissue sections were stained for optical microscope analysis (Nikon ECLipse Ci-L, Version 22.5 x64, Tokyo). Stained pictures were obtained using iSolution Lite (IMT i-Solution Inc., Vancouver, BC, Canada).

### 2.12. Calcification Analysis of the Scaffold After Implantation

For calcium quantification analysis of the harvested scaffolds, Micro-Computed Tomography (CT) analysis was conducted. Harvested scaffold samples from each group were rinsed with normal saline and then dried at RT for 5 min. CT images of each harvested sample were measured using a Micro-CT analyzer (Quantum GX II in vivo micro-CT, BR70000032-01, PerkinElmer), and the degree (volume of interest (VOI), mm^3^) of calcification in the images was analyzed by the Caliper Analyze 12.0 software program.

## 3. Results

### 3.1. Histological Characterization of Decellularized Porcine Pericardium

**Figure 1:**
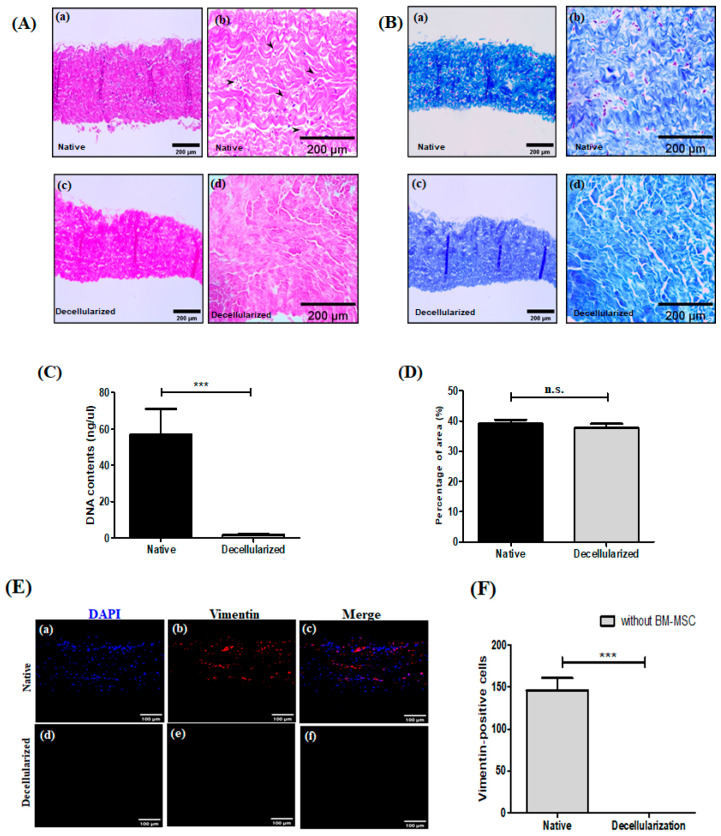
Histological, biochemical, and immunofluorescence evaluation in decellularized porcine pericardial scaffolds. (**A**) Hematoxylin and eosin (H&E) staining of native (a,b) and decellularized (c,d) porcine pericardium at ×200 and ×400 magnification. Arrowheads in (b) indicate cell nuclei present in the native tissue, which are absent in the decellularized group. (**B**) Masson’s trichrome staining of native (a,b) and decellularized (c,d) porcine pericardium at ×200 and ×400 magnification, showing preserved collagen fiber architecture in both groups. (**C**) Quantification of DNA content (ng/μL) in native and decellularized tissues. A significant reduction in DNA content is observed after decellularization (*** *p* < 0.001). Data are presented as the mean ± SD of three independent experiments. Statistical significance was calculated using one-way analysis of variance with an unpaired *t*-test. (**D**) Quantification of collagen deposition area (% area) in native and decellularized tissues, showing no significant difference (n.s.) between the groups. Data are presented as the mean ± SD of three independent experiments. Statistical significance was calculated using one-way analysis of variance with an unpaired *t*-test. (**E**) Representative immunofluorescence images showing nuclei stained with DAPI (blue, a,d), vimentin expression (red, b,e), and merged images (c,f) in native (top row) and decellularized (bottom row) scaffolds. A marked reduction in vimentin-positive cells is observed following decellularization. Magnification: ×200. (**F**) Quantification of vimentin-positive cells in native and decellularized tissues. A significant decrease in the number of vimentin-positive cells was observed in the decellularized group compared with the native tissue (*** *p* < 0.001). Data are presented as the mean ± SD of three independent experiments. Statistical significance was calculated using one-way analysis of variance with an unpaired *t*-test.

Porcine pericardial tissues were processed according to our developed decellularization protocol using 0.25% SDS + Triton X-100 and subjected to a multi-step method using hypotonic, isotonic, and hypertonic buffer solutions. The absence of cell nuclei was measured using hematoxylin-eosin staining and the DNA contents in the porcine pericardium after decellularization [Figure 1A,C]. The maintenance of ECM components was observed by histological staining with the Masson trichrome staining [Figure 1B] and the percentage area of collagen deposition [Figure 1D]. After decellularization, the removal of cell nuclear residues decreased compared with the native pericardium. In the histological characterization of the decellularized tissues, the collagen fibers maintained a structure that was not noticeably loosened and exhibited a distribution pattern similar to that of native tissue. As shown in Figure 1D, the quantified results of the percentage area of collagen deposition in the decellularized pericardium were not different compared with native pericardium. Thus, these results indicate that the decellularization process did affect the absence of DNA in pericardial tissues, but in contrast, did not affect collagen deposition in pericardial tissues.

In this study, we investigated the effects of decellularization on collagen organization. As shown in Figure 1B,D, the decellularized scaffolds exhibited collagen fiber distribution and deposition patterns similar to those of native tissue, and the structural integrity of the extracellular matrix was preserved after decellularization. To further evaluate the cellular content, we performed immunofluorescence staining using vimentin as an intercellular protein. As shown in Figure 1E, the vimentin expression of decellularized scaffolds decreased compared to native scaffolds. Quantitative analysis of vimentin expression revealed a decrease in the number of vimentin-positive cells in decellularized scaffolds [Figure 1F]. Thus, these results suggest that the decellularization process did affect the removal of vimentin.

### 3.2. In Vitro Evaluation of Recellularized Porcine Pericardium with ADSC Culture

#### 3.2.1. Histological Observation of ADSC Seeded onto Modified Pericardial Scaffold

**Figure 2:**
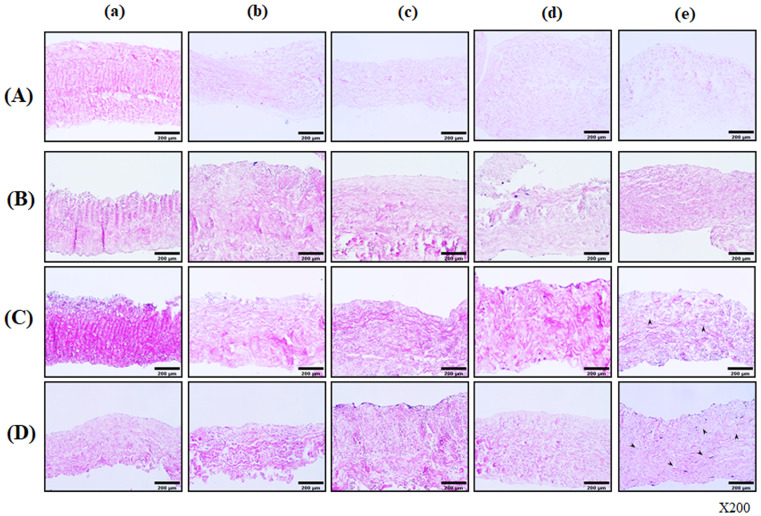
Histological characterization of Human ADSCs seeded onto fabricated pericardium. Histological observation of porcine pericardial scaffolds before and after cell seeding with hADSCs. The decellularized pericardial scaffolds were treated with or without 2000 unit/mL PNGase-F, respectively, in the absence and presence of α-galactosidase (0.1 unit/mL) for 24 h. Native (a), decellularized (b), decellularized-α-galactosidase (c), decellularized-PNGase-F (d), and decellularized-α-galactosidase/PNGase-F combination (e) scaffolds modified with a fibrin mesh, heparin and VEGF (Fb + H + VEGF). All modified scaffolds were seeded with hADSCs and were cultured for 7 d (**B**), 28 d (**C**), and 56 d (**D**). All modified pericardial scaffolds (**B**–**D**) were stained with H&E for comparison to the raw, uncultured scaffolds (0 d). An uncultured scaffold without cells was used as a negative control (**A**). By day 28, cell infiltration was most notable in the combination group (e), with deeper matrix penetration at day 56 (**D**-e, arrowheads). H&E stains were imaged using an optical microscope. The magnification used was ×200. arrowhead: nuclei.

**Figure 3:**
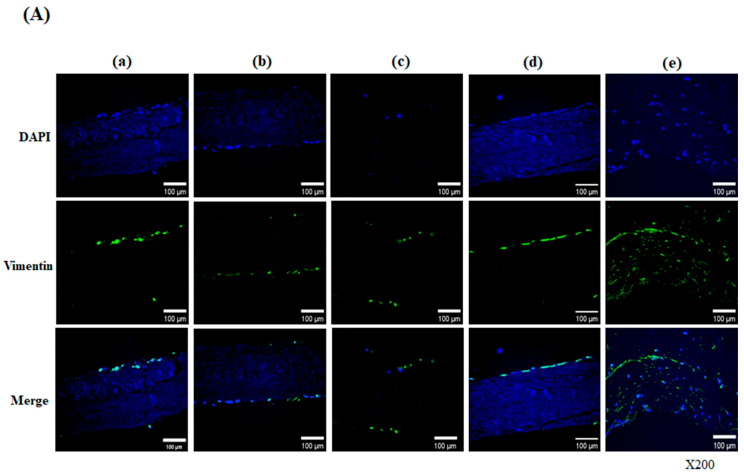

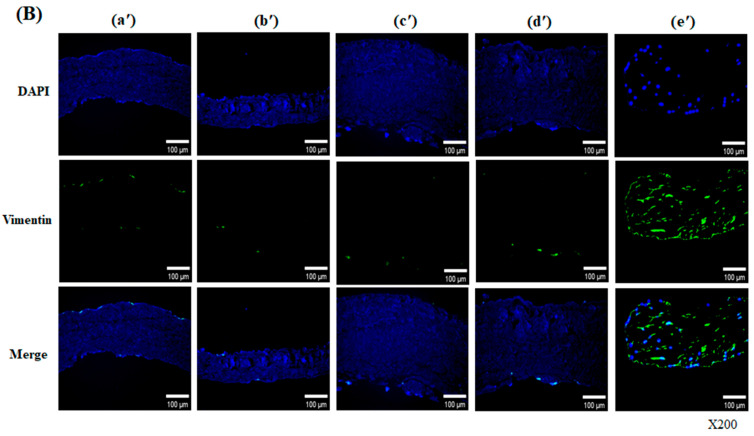
Vimentin expression in hADSC-seeded pericardial scaffolds on day 28 (**A**) and day 56 (**B**). Immunofluorescence images show DAPI (blue) and vimentin (green) staining in five scaffold groups: (a/a′) Native, (b/b′) Decellularized, (c/c′) +α-galactosidase, (d/d′) +PNGase-F, and (e/e′) +α-galactosidase/PNGase-F. Cells were stained with DAPI (blue) and vimentin (green). At day 28, surface staining was seen in most groups, while deep infiltration was found in the combination group (**A**-e). At day 56, strong and uniform vimentin expression was observed throughout the matrix in the combination group (**B**-e′). Magnification: ×200.

**Figure 4:**
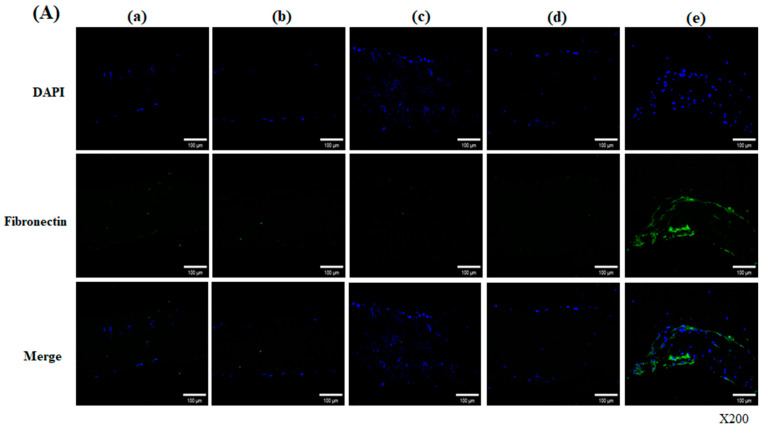

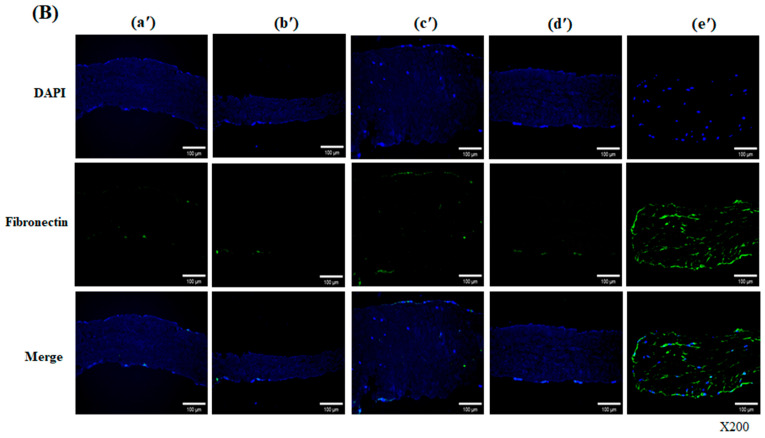
Fibronectin expression in hADSC-seeded pericardial scaffolds at day 28 (**A**) and day 56 (**B**). Immunofluorescence images show DAPI (blue) and fibronectin (green) staining in five scaffold groups: (a/a′) Native, (b/b′) Decellularized, (c/c′) +α-galactosidase, (d/d′) +PNGase-F, and (e/e′) +α-galactosidase/PNGase-F. Fibronectin expression was strongest in the combination-treated group (e,e′), especially on day 56, showing surface and matrix infiltration. Magnification: ×200.

**Figure 5:**
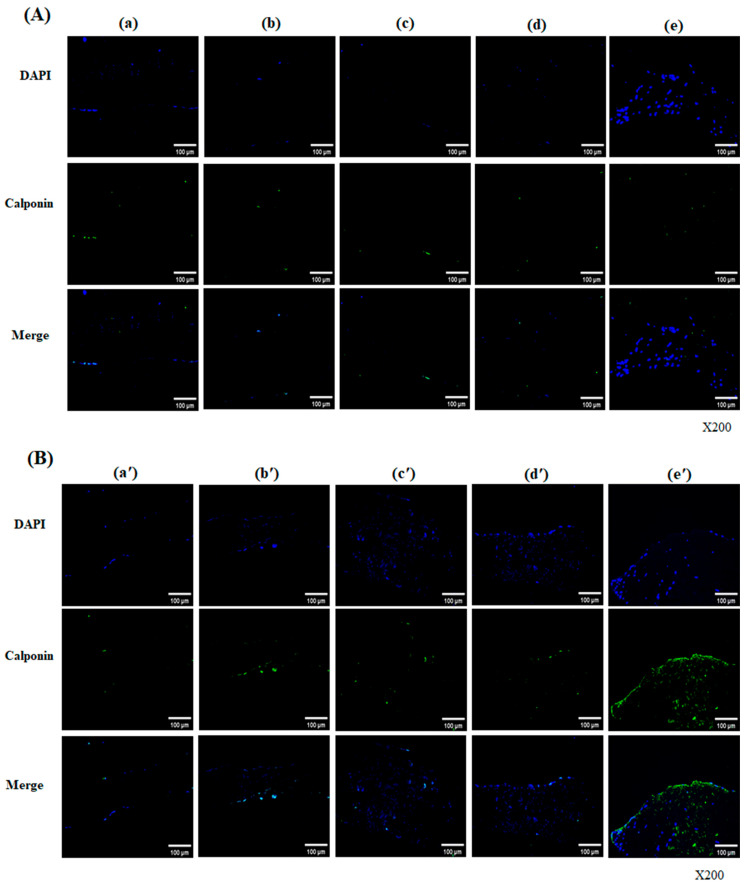
Calponin expression in hADSC-seeded pericardial scaffolds on day 28 (**A**) and day 56 (**B**). Immunofluorescence images show DAPI (blue) and calponin (green) staining in five scaffold groups: (a/a′) Native, (b/b′) Decellularized, (c/c′) +α-galactosidase, (d/d′) +PNGase-F, and (e/e′) +α-galactosidase/PNGase-F. Minimal calponin expression was seen on day 28 in all groups. By day 56, strong calponin signals were observed inside the matrix of the combination-treated group (e′), suggesting smooth muscle-like differentiation of hADSCs. Magnification: ×200.

**Figure 6:**
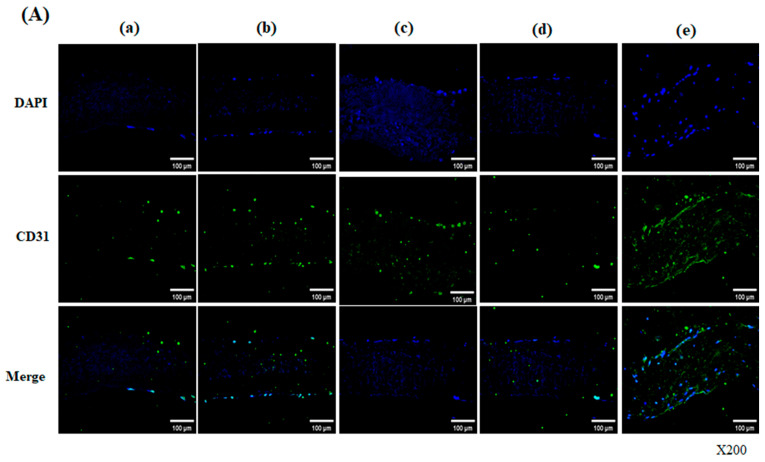

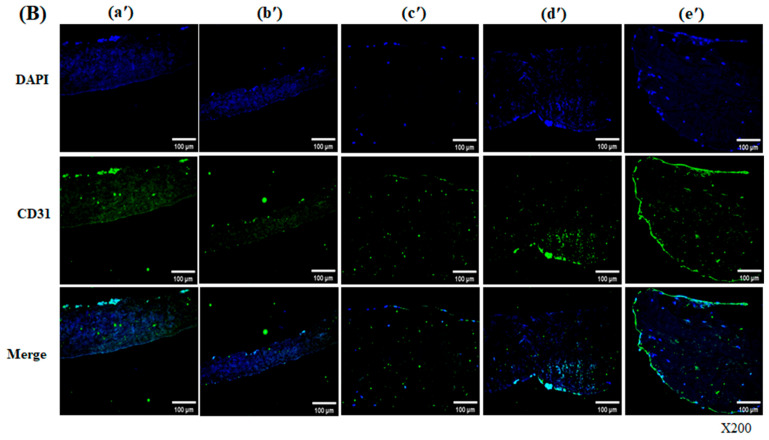
CD31 expression in hADSC-seeded pericardial scaffolds on day 28 (**A**) and day 56 (**B**). Immunofluorescence images of scaffolds treated under five conditions: (a/a′) Native, (b/b′) Decellularized, (c/c′) Decellularized + α-galactosidase, (d/d′) Decellularized + PNGase-F, (e/e′) Decellularized + α-galactosidase/PNGase-F combination. Scaffolds were seeded with hADSCs and cultured for 28 days (**A**) or 56 days (**B**), then stained for nuclei (DAPI, blue), CD31 (green), and merged (bottom rows). CD31-positive cells, indicating endothelial differentiation, were more abundant in the α-galactosidase/PNGase-F combination group (e,e′), especially on day 56. Magnification: ×200.

**Figure 7:**
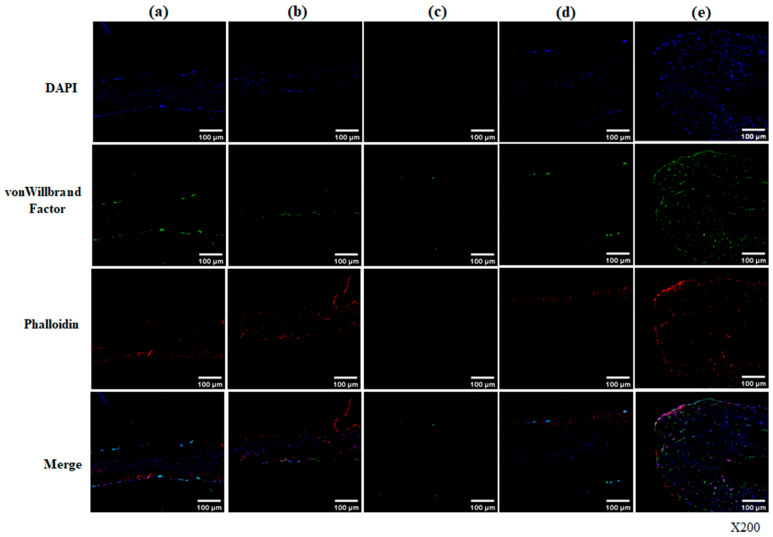
Confocal microscopy of endothelialization in hADSC-seeded pericardial scaffolds at day 56. Scaffolds were treated under five conditions: (**a**) Native, (**b**) Decellularized, (**c**) Decellularized + α-galactosidase, (**d**) Decellularized + PNGase-F, (**e**) Decellularized + α-galactosidase/PNGase-F combination. Tissues were stained for nuclei (DAPI, blue), von Willebrand factor (vWF, green), and phalloidin (red). Merged images show co-localization. In group (**e**), strong and widespread vWF and phalloidin signals were observed on the surface and throughout the matrix, indicating enhanced endothelialization by hADSCs. Magnification: ×100.

Recellularization of porcine pericardial scaffolds was achieved using hASDSs, with scaffold seeding and culturing performed in an ex vivo fashion. Native, decellularized, decellularized-α-galactosidase, decellularized-PNGase-F, and decellularized-α-galactosidase/PNGase-F combination pericardial scaffolds were coated with a fibrin mesh, heparin and VEGF (Fb + H + VEGF). Then, all coated pericardial scaffolds were seeded with hADSCs, cultured for 7, 28, and 56 days, and stained with H&E. On day 0, the uncultured modified scaffolds without cells were used as a negative control. After 28 days of cultivation, reseeded cells were predominantly found in the matrix of decellularized-α-galactosidase/PNGase-F-treated scaffolds [Figure 2C]. From day 56, decellularized-α-galactosidase/PNGase-F combination scaffolds were intensively infiltrated with hADSCs on the surface and inside the matrix. As shown in the representative image, a significant number of cells were observed to have penetrated deep into the pericardial scaffolds [Figure 2D(e)]. Cell infiltration can be distinguished by DAPI staining more clearly in the decellularized-α-galactosidase/PNGase-F combination than in other groups [Figure 3, Figure 4, Figure 5, Figure 6 and Figure 7].

#### 3.2.2. Immunofluorescence Staining in ADSCs Seeded onto Modified Pericardium

After fabrication, native, decellularized, decellularized-α-galactosidase, decellularized-PNGase-F, and decellularized-α-galactosidase/PNGase-F combination scaffolds were cultured for 28 days and 56 days, and the recellularization of these samples was visualized by immunofluorescence staining. The immunofluorescence analysis using various markers revealed cell infiltration, connective tissue remodeling, formation of new ECM components, and endothelialization in hADSCs seeded onto pericardial scaffolds following different treatments. Human ADSCs seeded onto pericardial scaffolds were found to express vimentin at day 28 and day 56 [Figure 3A,B]. Vimentin is an intermediate filament protein, mainly found in mesenchymal cells, that plays a role in cell migration, cell plasticity, and cellular organelle anchorage. Vimentin-positive cells penetrated intensively into the decellularized-α-galactosidase/PNGase-F samples, while in other samples, they were observed only partially on the surface. In the decellularized-α-galactosidase/PNGase-F combination scaffolds, vimentin-positive cells were shown to line the outer edge of the surface and penetrated partially into the tissue on day 28 [Figure 3A(e)]. From day 56, vimentin-positive cells were homogeneously present on the surface and inside the matrix of decellularized-α-galactosidase/PNGase-F combination scaffolds [Figure 3B(e′)].

Fibronectin is a major component of the ECM produced by several cell types that promotes cell adhesion, growth, migration, and differentiation. On day 28, stained samples revealed that fibronectin was partially retained within the decellularized-α-galactosidase/PNGase-F combination scaffolds, whereas it was not in the other groups [Figure 4A(e)]. From day 56, fibronectin-positive cells were predominantly observed on both the surface and inside matrix of the decellularized-α-galactosidase/PNGase-F combination scaffolds [Figure 4B(e′)].

Calponin is an actin filament-associated regulatory protein expressed in smooth muscle and non-muscle cells that is involved in regulating the contraction of smooth muscle contraction and cell differentiation into vascular smooth muscle cells. After 28 days of cultivation, there was minimal staining observed in all groups, but they partially showed an expression of calponin on the surface of the scaffolds at day 56 [Figure 5A,B]. In the decellularized-a-galactosidase/PNGase-F combination sample, calponin-positive signals were observed inside the tissue, suggesting that cells reseeded by hADSCs were found in the matrix of scaffolds, differentiating into smooth muscle cells [Figure 5B(e′)].

CD31 (Platelet Endothelial Cell Adhesion Molecule-1, PECAM-1) is an endothelial-specific marker with important roles in the endothelialization process. CD31 primarily mediates vascular angiogenesis, immune cell transmigration, and thrombogenesis. Immunofluorescence analysis of the hADSC-seeded scaffold section demonstrated an abundant thin monolayer of CD31-positive cells, indicating a regenerated endothelial differentiation on day 28 and day 56 [Figure 6A,B]. On day 28 and day 56, CD31-positive cells were observed to be homogeneously distributed on the surface of and inside the decellularized-α-galactosidase/PNGase-F-treated scaffolds, in contrast to other groups [Figure 6A(e′),B(e′)].

To explore the confocal microscopy for endothelialization in the hADSCs seeded onto scaffolds with fluorescently labeled phalloidin and von Willebrand factor (vWF) antibodies, we stained vWF and phalloidin in the scaffold on day 56. The vWF is a blood glycoprotein synthesized by endothelial cells that is required for normal hemostasis through platelet and subendothelial collagen adhesion and Factor VIII stabilization. Phalloidin is a specific bicyclic peptide used for staining the actin cytoskeleton in cells. As shown in Figure 7, the expression of vWF shows co-localization with phalloidin within the scaffold, particularly in the decellularized-α-gal/PNG-F combination scaffold. On day 56, confocal staining showed endothelialization by reseeded hADSCs, with VWF-positive cells (green) and phalloidin-positive cells (red) homogeneously distributed on the surface and inside the decellularized-α-galactosidase/PNGase-F combination scaffolds, in contrast to other groups [Figure 7e]. The endothelialization markers were broadly expressed throughout the decellularized-α-galactosidase/PNGase-F combination scaffolds repopulated by human ADSCs, indicating endothelialization forms in the recellularized graft.

### 3.3. In Vivo Evaluation of Recellularized Porcine Pericardial Scaffold with BM-MSC Culture

#### 3.3.1. Histological Observation of BM-MSCs Seeded Scaffolds Implanted into Rats

**Figure 8:**
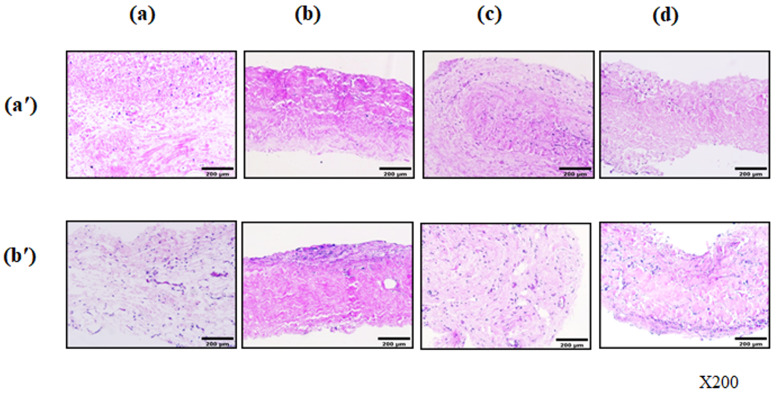
Histological analysis of BM-MSC-seeded pericardial scaffolds after implantation. Histological evaluation after scaffold implantation. Native (**a**), native-0.5% glutaraldehyde (GA) (**b**), decellularized (**c**), and decellularized–0.5% GA (**d**) scaffolds modified with a fibrin mesh, heparin and VEGF (Fb + H + VEGF). All modified scaffolds were seeded with rat BM-MSC (bone marrow mesenchymal stem cells) and were harvested at 7 d and 28 d after implantation. After harvest, all modified pericardial scaffolds were stained with H&E for comparison to the raw, native scaffolds. H&E stains were imaged using an optical microscope. All groups showed inflammatory cell infiltration. Inflammation was reduced in the native, decellularized, and decellularized + GA groups compared to the GA group at day 28. The upper image is of day 7 scaffolds (**a′**) and the lower image is of day 28 scaffolds (**b′**). The magnification used was ×200.

To further investigate the anti-calcification effect of recellularization on cross-linked scaffolds after the decellularization process, the anti-calcification effect of recellularization was performed in vivo. Porcine pericardial tissues cross-linked by the 0.5% glutaraldehyde method were explanted in rats to examine their calcification tendency and cellular integrity. Before being explanted, BM-MSCs were repopulated onto pericardial scaffolds. After implantation, the histological characterization of the harvested scaffolds was performed using H&E. H&E staining showed inflammatory cellular infiltrates within the scaffolds after implantation in all groups [Figure 8]. On day 28, the native, decellularized group, and decellularized–0.5% GA group had fewer inflammatory cells than the 0.5% GA group [Figure 8b′].

#### 3.3.2. Immunofluorescence Staining of BM-MSC-Seeded Scaffolds Implanted into Rats

**Figure 9:**
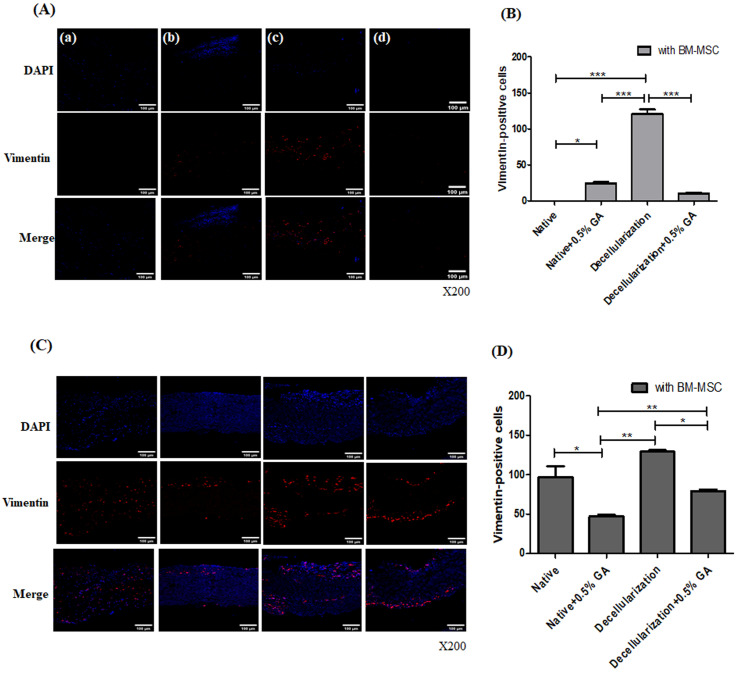
Immunofluorescence analysis of vimentin expression in BM-MSC seeded pericardial scaffolds after implantation (**A**,**C**). Immunofluorescence images of implanted scaffolds stained for nuclei (DAPI, blue) and vimentin (red) at day 7 (**A**) and day 28 (**C**). Groups: (a) Native, (b) Native + 0.5% GA, (c) Decellularized, (d) Decellularized + 0.5% GA. Strong vimentin expression was observed in the decellularized group, indicating enhanced cell infiltration. (**B**,**D**) Quantification of vimentin-positive cells at day 7 (**B**) and day 28 (**D**) showed significantly higher values in the decellularized group. Data are presented as the mean ± SD of five independent experiments. Statistical significance was calculated using one-way analysis of variance with Tukey’s multiple comparisons test. * *p* < 0.05, ** *p* < 0.01, *** *p* < 0.001. Magnification: ×200. * *p* < 0.05, ** *p* < 0.01, *** *p* < 0.001.

To determine whether recellularization restored cellular integrity on cross-linked scaffolds after decellularization with SDS-Tx, we examined the expression levels of vimentin. Native, native-0.5% GA, decellularized, and decellularized–0.5% GA harvested samples were stained with vimentin. On day 7, vimentin-positive cells were more prominently present on the surfaces of the decellularized groups, whereas fewer vimentin-positive cells were observed in the 0.5% GA groups and decellularized–0.5% GA groups [Figure 9A(c)]. By day 28, a significant number of cells were observed lining the surface and penetrating deeper into the tissue in the decellularized samples, with stronger vimentin signals compared to day 7 [Figure 9C(c)]. In addition, the decellularized group exhibited stronger vimentin expression than the other groups. Vimentin expression in the scaffolds was weaker in the 0.5% GA group than in the native group and decellularized–0.5% GA group. Moreover, a strong signal of vimentin was detected within the scaffold in the decellularized sample with cell seeding, exhibiting comparable signal intensity to that of the staining of native scaffolds (fresh control) without BM-MSC seeding [Figure 1E]. Quantitative analysis of vimentin expression revealed a significant increase in the number of vimentin-positive cells in decellularized scaffolds. As shown in Figure 9B, the number of vimentin-positive cells in the decellularized groups was higher than in the native groups, 0.5% GA groups, and decellularized–0.5% GA groups on day 7. Additionally, a large number of vimentin-positive cells were significantly higher on day 28 in decellularized groups than in native groups, 0.5% GA groups and decellularized–0.5 GA groups [Figure 9D]. In particular, the decellularized scaffolds showed faster mesenchymal stromal cell infiltration into the tissue, indicating an enhanced recellularization response in vivo.

#### 3.3.3. Calcium Staining and Calcium Levels of BM-MSCs Seeded Scaffold Implanted into Rats

**Figure 10:**
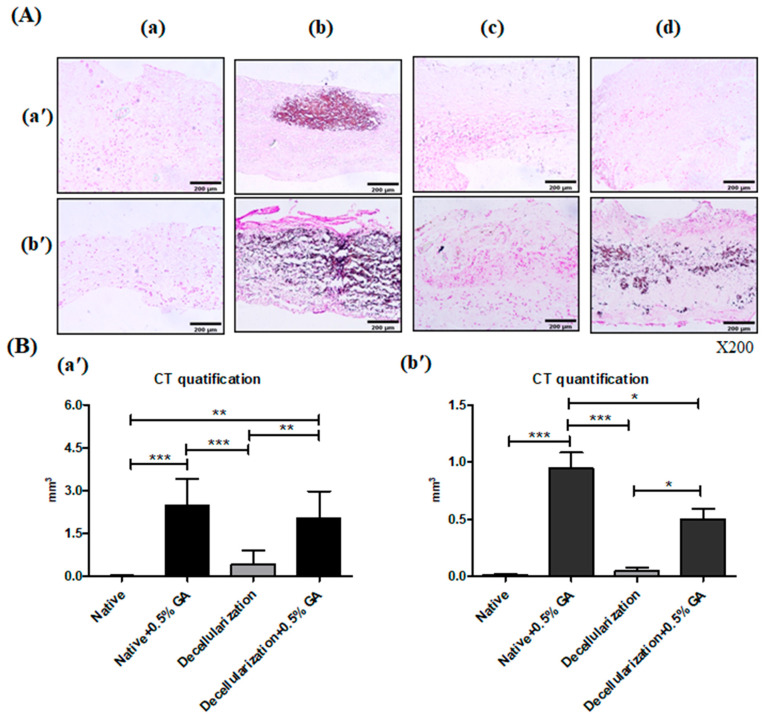
Assessment of calcification in the BM-MSC-seeded pericardial scaffolds after implantation. (**A**) Von Kossa staining of scaffold sections at day 7 (a′) and day 28 (b′). Groups: (a) Native, (b) Native + 0.5% GA, (c) Decellularized, (d) Decellularized + 0.5% GA. Extensive calcification was observed in the 0.5% GA group, while decellularized scaffolds showed reduced calcification, especially with BM-MSC repopulation. Magnification: ×200. (**B**) Micro-CT quantification of calcium deposits at day 28. Groups: (a′) acellular scaffolds (without BM-MSCs), (b′) MSC-seeded scaffolds (with BM-MSCs). Calcification volume was significantly higher in the 0.5% GA group without acellular scaffolds compared to other groups, and recellularization with MSC-seeded scaffolds (Decellularized + 0.5% GA) reduced calcium deposition. Graphs represent the mean ± SD values from seven independent experiments. Statistical significance was calculated using one-way analysis of variance with Tukey’s multiple comparisons test. * *p* < 0.05, ** *p* < 0.01, *** *p* < 0.001.

To determine whether recellularization reduces calcification in decellularized–glutaraldehyde (GA)-treated scaffolds after implantation, we repopulated BM-MSCs onto the modified scaffold after decellularization–0.5% GA treatment and assessed their calcium deposits through von Kossa staining. The calcification within scaffolds showed the results of in vivo experiments on rats with differently treated tissues implanted subcutaneously for 28 days (Figure 10A). As shown in Figure 10A(a′), calcification foci were present in the von Kossa stained image of the 0.5% GA group on day 7, whereas they were not found in the other groups. On day 28 after the implantation, staining revealed calcific deposits in the 0.5–GA group and decellularized–0.5% GA groups (Figure 10A(b′)). Moreover, the 0.5–GA group had more predominant calcific deposits than the decellularized–0.5% GA groups. Calcium depositions were weaker in the 0.5 GA groups with decellularization than in 0.5% GA groups without decellularization, indicating that recellularization by BM-MSC decreases calcific deposits compared to the non-recellularized tissue.

To confirm the anti-calcification effect of the recellularization on glutaraldehyde (GA)-treated scaffold after implantation, calcium deposits were quantified through Micro-CT measurements. Figure 10B shows the calcification volume in native, 0.5% GA, decellularization, and decellularized–0.5% GA, respectively. There was a significant difference in calcium quantification among the groups. As shown in Figure 10B(a′), in non-recellularized scaffolds, the calcification volume of the native groups (0.023 ± 0.023 mm^3^), decellularized groups (0.411 ± 0.474 mm^3^), and decellularized–0.5% GA groups (2.048 ± 0.927 mm^3^) were all lower than that of the 0.5% GA groups (2.487 ± 1.202 mm^3^, *p* < 0.01) on day 28. Figure 10B(b′) shows the measurements for calcium deposition on BM-MSC-seeded scaffolds on day 28, which were significantly higher in the 0.5% GA groups than in the decellularized–0.5% GA groups (0.942 ± 0.47 vs. 0.646 ± 0.294 mm^3^), while calcium depositions were not significantly increase in native groups (0.013 ± 0.01 mm^3^) and decellularized groups (0.048 ± 0.08 mm^3^) when compared with the 0.5% GA groups. In these results, the calcification volume of acellular scaffolds was significantly higher in the 0.5% GA groups compared to other groups, while the calcium deposition of recellularized scaffolds was significantly lower in the decellularized–0.5% GA groups than in the 0.5% GA group. The recellularized scaffolds exhibited a better anti-calcification performance than the non-recellularized GA-treated scaffolds, indicating that recellularization for BM-MSC reduced calcification compared to non-recellularized scaffolds.

## 4. Discussion

In this study, we successfully developed a clinically applicable recellularization strategy for decellularized and enzymatically xenoantigen-removed porcine pericardial scaffolds using hADSCs. Dual enzymatic removal of xenoantigens using α-Gal and PNGase-F significantly improved scaffold biocompatibility and promoted effective recellularization. Human ADSCs demonstrated infiltration, survival, and differentiation into valvular interstitial and endothelial-like phenotypes, as evidenced by expression of vimentin, fibronectin, calponin, CD31, and vWF. The functional integration of endothelial-like cells and ECM remodeling was further supported by phalloidin co-localization and progressive fibronectin expression. Furthermore, in vivo experiments confirmed that recellularization occurred within the biocompatible scaffolds after implantation, and a marked reduction in calcification was observed.

Importantly, the novelty of this study lies in several key aspects that distinguish it from prior work. While previous studies on xenogeneic valve recellularization have been limited by incomplete cell engraftment, reliance on non-human or mixed cell populations, and persistent xenoantigen-related immune responses, our approach addresses these challenges in an integrated manner. First, we established a xenoantigen-free scaffold through dual enzymatic removal of both α-Gal and non-α-Gal epitopes, thereby creating a biologically permissive environment for human cell engraftment. Second, we demonstrate, to the best of our knowledge, the first successful recellularization of porcine-derived cardiovascular tissue using hADSCs as a single-cell source without co-culture or pre-differentiation. Third, our strategy was directly applied to a clinically relevant xenograft platform, PULSTA, significantly enhancing its translational potential. Finally, by combining recellularization with a multi-step anti-calcification treatment, we extend the impact of recellularization beyond biocompatibility to improved resistance against structural degeneration, which remains a major limitation of current bioprosthetic valves.

To address the shortage of human-derived organs, the development of artificial or animal-derived tissues with excellent biocompatibility through tissue engineering has emerged as a particularly important issue, especially for patients with congenital heart disease. In our previous studies, we optimized the decellularization process to preserve the ECM structure and applied it to tissue valve construction [7]. However, to overcome the limitations of decellularized xenograft valves—particularly their deficiency in essential biological functions like regeneration, endothelialization, and immunomodulation—we shifted our focus toward strategies involving recellularization following decellularization [12,13]. We demonstrated that porcine xenograft tissue, after appropriate decellularization, could be recellularized in vitro by co-culturing hADSCs with HUVECs to accelerate the process [10]. To streamline the process for greater clinical feasibility, we explored a monoculture recellularization approach using adipose-derived stem cells alone.

Initially, our goal was to enhance the efficiency of recellularization using mesenchymal stem cells (MSCs) by modifying the surface of decellularized scaffolds, thereby addressing their functional limitations. Earlier studies have shown that integrating decellularized porcine aortic valves with porous matrix metalloproteinase (MMP) and degradable polyethylene glycol (PEG) hydrogels [14], or applying gelatin or fibronectin to the surface, resulted in improved cell attachment and proliferation [15]. As demonstrated in our previous study [10], we sought to improve MSC attachment and proliferation by incorporating heparin, fibrin mesh, and VEGF [11]. Fibrin-based modification can bind various growth factors and fibronectin present in the serum of the culture medium [16]. Additionally, fibronectin binding can be further enhanced through collagen-binding domains of the decellularized pericardium and through heparin conjugation [17], thereby promoting cell adhesion, proliferation, and migration.

Even if cells adhere to the surface of the scaffold, they may fail to engraft due to inflammatory immune responses, and calcification or thrombosis may occur first, leading to inflammation. Therefore, it is essential to sufficiently remove residual antigens from the xenogeneic tissue that can trigger inflammation. Notably, scaffolds treated with both α-galactosidase and PNGase-F showed superior recellularization outcomes compared to single enzyme or untreated controls, in addition to the decellularization strategy we have developed [2]. These findings highlight the synergistic role of dual enzyme pretreatment in reducing xeno-antigens and immunogenicity, thereby enhancing scaffold biocompatibility and enhancing cell adhesion and migration.

In addition, we used adipose-derived MSCs, which are relatively easy to obtain, for recellularization. Adipose-derived MSCs (AD-MSCs) and bone marrow-derived MSCs (BM-MSCs) are typically the most frequently utilized cell types for recellularization. Prior studies have indicated that these two MSC sources exhibit comparable levels of invasiveness, adhesion, and proliferation [18]. Moreover, MSCs exhibit distinct immunomodulatory properties, and research has demonstrated their ability to survive even in an allogenic environment [19]. If allogeneic MSCs can engraft and differentiate within xenogeneic tissues, they may retain their biological activity in a host environment. This potential has led to extensive research utilizing MSCs in tissue engineering.

Interestingly, stem cell proliferation and differentiation are regulated by interactions with the extracellular matrix and the surrounding culture environment. Thus, decellularized tissue scaffolds can serve as a key signaling platform guiding cell fate decisions. A previous study investigated the chondrogenic differentiation of bovine-derived MSCs when cultured in a collagen type II-rich environment [20]. Given that porcine pericardial tissue was used in our study, we anticipated that the seeded stem cells would interact with the residual ECM components and differentiate into cell types characteristic of cardiac or valvular tissue. It has been shown that decellularized rat-derived cardiac ECM can promote the recellularization of human embryonic stem cell-derived cardiomyocytes [21]. In another study of Fernanda et al., atrial-derived ECM was found to direct human iPSC-derived cardiomyocytes toward an atrial-specific phenotype [22]. These results indicate that residual ECM components within the scaffold play a critical role in guiding the acquisition of mature phenotypes in multipotent cells. Although this study did not determine the specific ECM components that remained or the signaling pathways driving stem cell differentiation, immunohistochemical staining revealed the potential for differentiation into valvular interstitial cells or valvular endothelial cells. Therefore, it is crucial to preserve the structural and biochemical integrity of the ECM during the cell removal process, as well as to remove it effectively.

In vitro culture of hADSC-seeded decellularized scaffolds demonstrated progressive recellularization and phenotypic differentiation. Vimentin-positive cells infiltrated the scaffold by day 28, with widespread distribution by day 56. Particularly, α-galactosidase/PNGase F-treated scaffolds showed enhanced vimentin-positive cell penetration, indicating effective recellularization. Fibronectin, a key ECM protein involved in cell adhesion, was initially weakly expressed at day 28 but became prominently localized on the surface and within the matrix by day 56. This temporal increase suggests that MSCs not only adhered and survived on the scaffold but also actively participated in ECM remodeling. While some fibronectin may have originated from residual native ECM, the increased expression over time implies new synthesis by hADSCs. This aligns with previous findings by Lee et al., who reported that fibronectin-derived peptides promote stem cell adhesion, proliferation, and differentiation via integrin-mediated signaling [23]. Calponin expression within the scaffold matrix suggests early differentiation of hADSCs toward smooth muscle-like cells, potentially supporting vascular remodeling. Although markers such as α-SMA, actin/myosin filaments, and collagen types I and III were not assessed, the positive expression of calponin and vimentin indicates that the ADSCs likely differentiated into myofibroblast-like cells, reflecting the contractile property and ECM-producing capability required in heart valve tissue [24].

Endothelial differentiation was evidenced by the presence of CD31- and von Willebrand factor (vWF)-positive cells forming a monolayer across the scaffold surface. These cells mimic native endothelial function, acting as a barrier to blood components and contributing to immunomodulation and thromboresistance. Endothelial cells additionally release a range of growth factors and signaling molecules that support the regeneration of surrounding tissue and the remodeling of the ECM, forming a basis for self-remodeling [25]. Importantly, vWF and phalloidin co-expression indicated that the differentiated cells not only acquired endothelial identity but also developed a well-organized cytoskeletal structure, suggesting a stable, functionally active state [26,27]. This co-expression was observed not only on the surface but also within the scaffold, demonstrating integration and migration of endothelial-like cells throughout the construct. This implies both functional endothelial differentiation and structural stability, both of which are crucial factors in tissue engineering applications.

To investigate the functional relevance of recellularization in vivo, we implanted BM-MSC–seeded scaffolds into subcutaneous pockets in rats. Histological analysis revealed increased vimentin expression and greater cell infiltration in the decellularized groups, particularly in scaffolds without GA fixation. Furthermore, von Kossa staining and Micro-CT analysis demonstrated reduced calcification in recellularized biocompatible scaffolds compared to GA-treated scaffolds. These results indicate that recellularization promotes cell integration and ECM remodeling while also reducing the risk of calcification- a critical drawback of GA-fixed bioprosthetic valves. We have recognized that xenogeneic tissues tend to lose durability due to calcification after implantation in the human body, and we have been studying methods to reduce this issue. We developed a four-step anti-calcification protocol—decellularization [4], space filling, organic solvent treatment, and detoxification—to reduce immunogenicity, prevent calcification, and improve the durability of xenograft tissues for cardiovascular use [28]. GA treatment has been widely used to enhance the durability of decellularized heart valve tissues. However, residual aldehyde groups left after GA fixation can induce cytotoxicity, inhibit endothelialization, and accelerate valve dysfunction by promoting calcification, thrombosis, and chronic inflammation [29,30]. In this study, even when MSCs were seeded onto GA-fixed tissues, in vivo analysis showed that recellularization of the scaffold occurred. Notably, the recellularized tissues exhibited the lowest levels of calcification, which was quantitatively confirmed. Although further studies are needed to determine whether the recellularized tissue can maintain durability and support ongoing cell survival and remodeling in vivo, it is encouraging that recellularization was achievable even after GA fixation, leading to successful cell engraftment and reduced calcification.

This study strongly supports that stem cell-based recellularization can produce functionally viable tissue grafts, particularly when combined with effective xenoantigen removal, and also demonstrates the potential of recellularization to enhance anti-calcification properties. However, we were limited by the inability to quantitatively assess the extent of immunostaining and did not include gene expression data as additional indicators [24]. While our findings provide that recellularization can reduce the degree of calcification, further research is required to clarify how the recellularized tissue responds in vivo, especially in terms of its immunogenic profile and the potential for sustained histological remodeling. Furthermore, it remains unclear to what extent the recellularized valve may induce inflammation upon in vivo implantation, and whether this inflammatory response could ultimately promote an M2-mediated immune reaction leading to long-term tissue healing and regeneration [13]. According to previous studies, heart valves recellularized with MSCs showed increased expression of CD163, a marker of M2 macrophages [31], which is associated with an anti-inflammatory and pro-regenerative environment. Recellularized xenogeneic valves using autologous mesenchymal stem cells showed a marked reduction in xenoreactive immune response, with immune cell infiltration and immunoglobulin production levels comparable to those of autologous tissue [32]. In our next study, we plan to further investigate the immune response in vivo. It has been demonstrated that applying biomimetic flow conditions to tissue-engineered constructs in vitro promotes tissue maturation and enhances mechanical properties. This study underscores the need for further investigation to assess whether the recellularized xenogeneic tissue can preserve its structural integrity and facilitate tissue development and cellular differentiation under biomimetic conditions.

This study has several limitations that should be considered in light of its defined scope. First, the analysis was primarily focused on early-stage recellularization and endothelial-like differentiation based on structural and phenotypic markers. Accordingly, detailed characterization of immune responses, macrophage involvement, and endothelial activation was not included. Even at early stages of differentiation, endothelial-like cell development is inherently associated with inflammatory and immune processes, particularly involving macrophage activity and smooth muscle cell (SMC) behavior. In particular, macrophage polarization—especially toward an M2 phenotype—plays a critical role in inducing the contractile phenotype of SMCs. These contractile SMCs provide a necessary substrate for endothelial cell adhesion, as endothelial-like cells are unlikely to directly attach to decellularized valve matrices in the absence of an underlying SMC layer. Furthermore, M2 macrophages and contractile SMCs are known to act synergistically to promote the formation of a stable endothelial monolayer, which is essential for sustained antithrombogenic function. In this context, markers such as CD86/CD206 for macrophage polarization, SM22α and osteopontin for SMC phenotypic switching, and E-selectin for endothelial activation, as well as functional assays including nitric oxide production, would provide a more comprehensive evaluation of these coordinated processes.

Second, endothelialization in this study was assessed mainly through established marker expression without comprehensive functional validation. While additional functional assays and advanced experimental models—such as biomimetic flow systems, long-term culture, and in vivo functional studies—would allow for a more detailed investigation of immune–mechanical interactions and endothelial functionality, these aspects were beyond the primary objectives of the present work. Importantly, although these mechanistic pathways were not directly evaluated, the in vivo biocompatibility and calcification findings provide supportive, albeit indirect, evidence of a favorable host response and tissue integration. The observed reduction in calcification and enhanced cellular incorporation suggest the establishment of a microenvironment that may be permissive to macrophage-mediated modulation and subsequent SMC and endothelial organization.

Finally, given that vascular remodeling and endothelialization are temporally regulated, multicellular processes involving coordinated interactions among macrophages, smooth muscle cells, and endothelial cells, the present findings should be interpreted as reflecting an early stage of tissue development rather than a fully matured or functionally complete tissue. Future studies incorporating targeted analyses of macrophage polarization, SMC contractility, and endothelial functionality under physiologically relevant conditions will be essential to further elucidate these mechanisms.

In summary, our dual enzyme treatment significantly enhanced scaffold biocompatibility and hADSC-mediated recellularization. Vimentin and fibronectin expression confirmed interstitial-like cell phenotypes and ECM remodeling, while CD31 and vWF expression with phalloidin co-localization indicated successful endothelial differentiation and cytoskeletal organization. In vivo, recellularized biocompatible scaffolds exhibited increased cell infiltration and markedly reduced calcification compared to GA-fixed controls. Our hADSC-based monoculture recellularization, combined with optimized xenoantigen removal, effectively supports functional repopulation and reduces calcification in porcine pericardial scaffolds. This approach offers a simplified and scalable path toward developing next-generation, recellularized tissue-engineered heart valves.

## 5. Conclusions

We demonstrated that the combination of dual enzyme-mediated xenoantigen removal and hADSC recellularization effectively promotes cell infiltration, differentiation, endothelialization, and anti-calcification in porcine pericardial scaffolds for the first time in the world. These results suggest that the integration of optimized antigen removal and single-cell lineage recellularization may enable the development of biocompatible and functional tissue-engineered grafts. Thus, the tissue engineering by recellularizing and remodeling decellularized xenogeneic scaffolds using MSCs appears to be a promising candidate for next-generation heart valve tissue. It holds the potential to reduce calcification, promote rapid endothelialization and cellular differentiation, and ultimately undergo remodeling after implantation in vivo.

## Data Availability

The original contributions presented in this study are included in the article. Further inquiries can be directed to the corresponding author.

## References

[1] Mendelson K., Schoen F.J. (2006). Heart Valve Tissue Engineering: Concepts, Approaches, Progress, and Challenges. Ann. Biomed. Eng..

[2] Kim S.Y., Kim G.B., Lim H.G., Kim Y.J. (2024). Strategies beyond Decellularization for Optimal Tissue Engineering of Cardiac Xenografts. J. Biomed. Res. Environ. Sci..

[3] Kim W.G., Park J.K., Lee W.Y. (2002). Tissue-Engineered Heart Valve Leaflets: An Effective Method of Obtaining Acellularized Valve Xenografts. Int. J. Artif. Organs.

[4] Lim H.G., Kim S.H., Choi S.Y., Kim Y.J. (2012). Anticalcification effects of decellularization, solvent, and detoxification treatment for genipin and glutaraldehyde fixation of bovine pericardium. Eur. J. Cardio-Thorac. Surg..

[5] Lim H.G., Kim G.B., Jeong S., Kim Y.J. (2015). Development of a next-generation tissue valve using a glutaraldehyde-fixed porcine aortic valve treated with decellularization, α-galactosidase, space filler, organic solvent and detoxification. Eur. J. Cardio-Thorac. Surg..

[6] Lim H.G., Choi S.Y., Yoon E.J., Kim S.H., Kim Y.J. (2013). In Vivo Efficacy of Alpha-Galactosidase as Possible Promise for Prolonged Durability of Bioprosthetic Heart Valve Using Alpha1,3-Galactosyltransferase Knockout Mouse. Tissue Eng. Part A.

[7] Kim G.B., Kwon B.S., Lim H.G. (2017). First in human experience of a new self-expandable percutaneous pulmonary valve implantation using knitted nitinol-wire and tri-leaflet porcine pericardial valve in the native right ventricular outflow tract. Catheter. Cardiovasc. Interv..

[8] Kim G.B., Lim H.G., Kim Y.J., Choi E.Y., Kwon B.S., Jeong S. (2014). Novel self-expandable, stent-based transcatheter pulmonic valve: A preclinical animal study. Int. J. Cardiol..

[9] Kim G.B., Song M.K., Bae E.J., Park E.-A., Lee W., Lim H.-G., Kim Y.J. (2018). Successful Feasibility Human Trial of a New Self-Expandable Percutaneous Pulmonary Valve (Pulsta Valve) Implantation Using Knitted Nitinol Wire Backbone and Trileaflet α-Gal–Free Porcine Pericardial Valve in the Native Right Ventricular Outflow Tract. Circ. Cardiovasc. Interv..

[10] Yoon J.K., Kim S.Y., Kim S., Lee K.M., Ko S., Kim G.B., Lim H.G., Kim Y.J. (2025). Human recellularization for xenoantigen free decellularized cardiac xenografts. Tissue Eng. Part A.

[11] Filova E., Steinerova M., Travnickova M., Knitlova J., Musilkova J., Eckhardt A., Hadraba D., Matejka R., Prazak S., Stepanovska J. (2021). Accelerated in vitro recellularization of decellularized porcine pericardium for cardiovascular grafts. Biomed. Mater..

[12] Syedain Z.H., Bradee A.R., Kren S., Taylor D.A., Tranquillo R.T. (2013). Decellularized Tissue-Engineered Heart Valve Leaflets with Recellularization Potential. Tissue Eng. Part A.

[13] Fioretta E.S., Motta S.E., Lintas V., Loerakker S., Parker K.K., Baaijens F.P.T., Falk V., Hoerstrup S.P., Emmert M.Y. (2021). Next-generation tissue-engineered heart valves with repair, remodelling and regeneration capacity. Nat. Rev. Cardiol..

[14] Dai J., Qiao W., Shi J., Liu C., Hu X., Dong N. (2019). Modifying decellularized aortic valve scaffolds with stromal cell-derived factor-1α loaded proteolytically degradable hydrogel for recellularization and remodeling. Acta Biomater..

[15] Sugimura Y., Chekhoeva A., Oyama K., Nakanishi S., Toshmatova M., Miyahara S., Barth M., Assmann A.K., Lichtenberg A., Assmann A. (2020). Controlled autologous recellularization and inhibited degeneration of decellularized vascular implants by side-specific coating with stromal cell-derived factor 1 and fibronectin. Biomed. Mater..

[16] Riedelová-Reicheltová Z., Brynda E., Riedel T. (2016). Fibrin Nanostructures for Biomedical Applications. Physiol. Res..

[17] Martino M.M., Briquez P.S., Ranga A., Lutolf M.P., Hubbell J.A. (2013). Heparin-binding domain of fibrin(ogen) binds growth factors and promotes tissue repair when incorporated within a synthetic matrix. Proc. Natl. Acad. Sci. USA.

[18] Bonvillain R.W., Danchuk S., Sullivan D.E., Betancourt A.M., Semon J.A., Eagle M.E., Mayeux J.P., Gregory A.N., Wang G., Townley I.K. (2012). A Nonhuman Primate Model of Lung Regeneration: Detergent-Mediated Decellularization and Initial In Vitro Recellularization with Mesenchymal Stem Cells. Tissue Eng. Part A.

[19] Liechty K.W., MacKenzie T.C., Shaaban A.F., Radu A., Moseley A.B., Deans R., Marshak D.R., Flake A.W. (2000). Human mesenchymal stem cells engraft and demonstrate site-specific differentiation after in utero transplantation in sheep. Nat. Med..

[20] Bosnakovski D., Mizuno M., Kim G., Takagi S., Okumura M., Fujinaga T. (2006). Chondrogenic differentiation of bovine bone marrow mesenchymal stem cells (MSCs) in different hydrogels: Influence of collagen type II extracellular matrix on MSC chondrogenesis. Biotechnol. Bioeng..

[21] Hochman-Mendez C., Campos D.B.P.d., Pinto R.S., Mendes B.J.d.S., Rocha G.M., Monnerat G., Weissmuller G., Sampaio L.C., Carvalho A.B., A Taylor D. (2020). Tissue-engineered human embryonic stem cell-containing cardiac patches: Evaluating recellularization of decellularized matrix. J. Tissue Eng..

[22] Mesquita F.C.P., Morrissey J., Lee P.-F., Monnerat G., Xi Y., Andersson H., Nogueira F.C.S., Domont G.B., Sampaio L.C., Hochman-Mendez C. (2021). Cues from human atrial extracellular matrix enrich the atrial differentiation of human induced pluripotent stem cell-derived cardiomyocytes. Biomater. Sci..

[23] Lee E.-J., Ahmad K., Pathak S., Lee S., Baig M.H., Jeong J.-H., Doh K.-O., Lee D.-M., Choi I. (2021). Identification of Novel FNIN2 and FNIN3 Fibronectin-Derived Peptides That Promote Cell Adhesion, Proliferation and Differentiation in Primary Cells and Stem Cells. Int. J. Mol. Sci..

[24] Hoerstrup S.P., Kadner A., Melnitchouk S., Trojan A., Eid K., Tracy J., Sodian R., Visjager J.F., Kolb S.A., Grunenfelder J. (2002). Tissue engineering of functional trileaflet heart valves from human marrow stromal cells. Circulation.

[25] Motta S.E., Zaytseva P., Fioretta E.S., Lintas V., Breymann C., Hoerstrup S.P., Emmert M.Y. (2022). Endothelial Progenitor Cell-Based in vitro Pre-Endothelialization of Human Cell-Derived Biomimetic Regenerative Matrices for Next-Generation Transcatheter Heart Valves Applications. Front. Bioeng. Biotechnol..

[26] Shojaei S., Tafazzoli-Shadpour M., Shokrgozar M.A., Haghighipour N., Jahromi F.H. (2018). Stress phase angle regulates differentiation of human adipose-derived stem cells toward endothelial phenotype. Prog. Biomater..

[27] Summer S., Rossmanith E., Pasztorek M., Fiedler C., Gröger M., Rauscher S., Weber V., Fischer M.B. (2022). Mesenchymal stem cells support human vascular endothelial cells to form vascular sprouts in human platelet lysate-based matrices. PLoS ONE.

[28] Lim H.G., Lee C.H., Lim J.H., Kim E.R., Kim Y.J. (2025). Clinical Results for Cardiovascular Xenografts Treated With Novel Anticalcification Protocols. Artif. Organs.

[29] Li J., Qiao W., Liu Y., Lei H., Wang S., Xu Y., Zhou Y., Wen S., Yang Z., Wan W. (2024). Facile engineering of interactive double network hydrogels for heart valve regeneration. Nat. Commun..

[30] Schoen F.J., Levy R.J. (2005). Calcification of Tissue Heart Valve Substitutes: Progress Toward Understanding and Prevention. Ann. Thorac. Surg..

[31] Khorramirouz R., Go J.L., Noble C., Morse D., Lerman A., Young M.D. (2019). In Vivo Response of Acellular Porcine Pericardial for Tissue Engineered Transcatheter Aortic Valves. Sci. Rep..

[32] Bozso S.J., Kang J.J.H., EL-Andari R., Fialka N., Zhu L.F., Meyer S.R., Freed D.H., Nagendran J., Nagendran J. (2022). Recellularization of xenograft heart valves reduces the xenoreactive immune response in an in vivo rat model. Eur. J. Cardio-Thorac. Surg..

